# Climbing the ladder of confidence: effects of the step ladder system on medical students’ self-efficacy during a surgical clerkship

**DOI:** 10.1186/s12909-026-09110-0

**Published:** 2026-04-06

**Authors:** Takehiko Hanaki, Rumiko Omatsu, Kozo Miyatani, Kyoichi Kihara, Hiroaki Komatsu, Yoshiyuki Fujiwara, Masaru Ueki

**Affiliations:** 1 Division of Medical Education, Tottori University Faculty of Medicine, Yonago, Japan; 2 Division of Gastrointestinal and Pediatric Surgery, Tottori University Faculty of Medicine, Yonago, Japan; 3 Division of Urology, Tottori University Faculty of Medicine, Yonago, Japan; 4 Division of Obstetrics and Gynecology, Tottori University Faculty of Medicine, Yonago, Japan

**Keywords:** General Self-Efficacy Scale (GSES), Step Ladder System (SLS), Clinical Clerkship, Surgical Clerkship, Medical Students

## Abstract

**Background:**

Self-efficacy is a key factor in motivation, persistence, and performance in medical education. However, surgical clerkships often provide limited hands-on practice and inconsistent feedback, hindering students’ ability to recognize their learning progress or readiness. The Step Ladder System (SLS), a structured competency-based framework, was introduced to clarify expectations and support incremental learning by helping students visualize progress and document task achievements. This study examined changes in medical students’ self-efficacy during a surgical clerkship and investigated whether participation in the SLS was linked to self-efficacy growth.

**Methods:**

An observational study using prospective and retrospective data was conducted among 24 sixth-year medical students who completed a four-week clerkship in gastroentintestinal and pediatric surgery at Tottori University in 2025. Self-efficacy was measured with the General Self-Efficacy Scale (GSES) at the start and end of the rotation. SLS engagement was calculated as the total number of task entries submitted via the mobile app. Paired t-tests evaluated pre- and post-rotation changes, while binomial tests analyzed directional trends in ΔGSES.

**Results:**

Standardized GSES scores increased significantly from pre- to post-clerkship (49.8 ± 11.0 to 53.8 ± 12.2; *p =* 0.009). Students with high SLS engagement were more likely to show positive rather than negative ΔGSES (*p =* 0.021), while no clear trend was observed in low-engagement students. Learners with lower baseline GSES showed a significant predominance of positive ΔGSES (*p =* 0.031).

**Conclusions:**

Medical students’ self-efficacy improved over the clerkship. The SLS is designed as a learner-facing scaffold that supports clarification of expectations and structured preparation, rather than a tool for summative competency assessment. The SLS may support this growth by clarifying expected learning targets and expectations, encouraging purposeful preparation, and providing clearer goals and structured mastery experiences. Although SLS engagement and the magnitude of ΔGSES were not proportional, greater engagement and lower initial self-efficacy were consistently associated with positive change, suggesting that structured task engagement influences self-efficacy through non-linear mechanisms. The SLS appears to be a feasible and potentially beneficial framework for learner-centered surgical education.

**Supplementary Information:**

The online version contains supplementary material available at 10.1186/s12909-026-09110-0.

## Background

Within the field of educational psychology, self-efficacy denotes a person’s confidence in being able to structure and implement the behaviors required to reach intended goals, and it plays a fundamental role in shaping motivation and achievement [1, 2]. In medical education, higher self-efficacy has been linked to stronger learning motivation, active engagement, and perceived clinical performance [3]. Conversely, low self-efficacy often leads to anxiety, passivity, and poor skill retention [4, 5]. Strengthening self-efficacy during clinical clerkships is therefore a key goal within competency-based medical education [6, 7].

However, surgical clerkships present unique challenges to fostering self-efficacy. Students are frequently placed in observer roles within hierarchical surgical teams, with limited hands-on opportunities and inconsistent feedback [8, 9]. These factors can obscure learners’ sense of progress and competence, making it difficult for them to recognize skill development or internalize achievement. Even after completing clerkships, many students remain uncertain about their capabilities and readiness for clinical practice [10, 11].

To address these educational challenges, Komatsu et al. developed the Step Ladder System (SLS), a structured learning support framework designed to visualize expected competencies and support self-directed and reflective learning in medical education [12]. The SLS organizes core clinical competencies into tiered levels of mastery, guiding learners from observation through assistance to independent performance. By documenting experiences and reflecting on progress, students can visualize their growth while instructors can monitor engagement and provide targeted feedback [12]. Previous reports have suggested that such structured visualization of competency progression enhances learning motivation and reflective practice [13, 14]. In addition to learner-focused outcomes, ladder-based competency frameworks may also influence educational behaviors among supervising physicians. Arai et al. reported that clinical clerkships integrated with a clinical ladder improved teaching self-efficacy among attending physicians, particularly those with less teaching experience [15]. Although their findings concern educators rather than students, the study highlights that tiered competency structures can enhance clarity of expectations and promote more deliberate engagement in clinical teaching. Such mechanisms may also support psychological development among learners; however, empirical evidence on student self-efficacy in surgical clerkships remains limited. Taken together, prior evidence supports the educational value of tiered competency frameworks and provides a rationale for implementing such systems in surgical clerkships, where structured guidance and consistent feedback are often limited.

Against this background, Tottori University expanded the SLS to include the surgical clerkship curriculum in the Division of Gastrointestinal and Pediatric Surgery starting in 2025. Within this framework, students document both procedural experiences and knowledge-related achievements, such as understanding key anatomical concepts, principles of perioperative management, and disease-specific decision-making. This allows for the creation of a personalized, step-by-step visualization of both technical progress and cognitive development. Despite increasing interest in competency-based frameworks, there is limited evidence on how task-based engagement within such systems impacts medical students’ self-efficacy during surgical clerkships. It remains unclear whether a structured, tiered competency tool can effectively support psychological growth in a rotation that traditionally offers limited feedback and learner autonomy. This study addresses this gap by examining changes in self-efficacy and their relationship with engagement in the Step Ladder System. The goal of this study was to assess whether the SLS supports students’ perceived readiness and confidence for learning during surgical clerkships by analyzing changes in the General Self-Efficacy Scale (GSES) scores [16, 17] and exploring the relationship between SLS engagement and self-efficacy development.

## Methods

### Data collection procedures

For students who completed the clerkship between April and May 2025, the GSES had already been administered as part of routine educational evaluation at both the start and end of the clerkship. These pre-existing GSES scores were retrospectively included in the analysis with institutional review board approval, and students were given an opt-out option to request withdrawal of their data.

Starting from June 2025, data were collected prospectively. During the orientation session on the first day of the clerkship, all students received written information about the study and provided written informed consent. The GSES was administered at two points: the first day (pre-test) and the final day (post-test) of the four-week rotation. All questionnaires were completed independently in a controlled environment that ensured privacy.

For both the retrospective and prospective parts, each student’s GSES responses were linked with an anonymous alphanumeric identification code. The linkage table was kept separate from the questionnaires, and all datasets used for analysis were fully anonymized before use.

### Participants

All sixth-year medical students assigned to the four-week elective clerkship in the Division of Gastrointestinal and Pediatric Surgery between April and October 2025 were eligible for inclusion. During this period, a total of 24 students participated, with four students in each cycle. Students were divided into four subspecialty groups: upper gastrointestinal, lower gastrointestinal, hepatobiliary-pancreatic, and pediatric surgery, following the department’s standard allocation procedures. Each student was supervised by a primary attending surgeon, with additional clinical teaching provided collaboratively by other members of the surgical team.

For students who completed the clerkship between April and May 2025, GSES assessments were conducted as part of routine educational evaluation, and these data were retrospectively included with approval from the institutional review board. An opt-out mechanism was provided to give students the opportunity to withdraw their data.

Starting in June 2025, all eligible students received a written explanation of the study's purpose, procedures, and data handling, and were informed that participation was completely voluntary and would not impact their clerkship evaluations, learning opportunities, or academic standing. Written informed consent was obtained from all students in the prospective cohort. No exclusion criteria were used.

### Educational intervention: Step Ladder System (SLS)

During the clerkship, all students used the SLS, a structured, competency-based learning framework designed to promote self-directed preparation and progressive skill development. The SLS includes multiple achievable clinical tasks arranged into a three-tier hierarchy that reflects increasing levels of difficulty and independence within gastrointestinal and pediatric surgery. The number and complexity of tasks were carefully calibrated so that a first- or second-year resident (Post Graduate Year 1–2) could reasonably complete all items after about three months of training, ensuring that the task set would remain manageable for undergraduate students while still representing authentic clinical competencies [18].

Prior to the clerkship, students could review the SLS tasks and identify the essential knowledge and procedures expected of them. This advanced visibility of learning objectives is a core feature of the system and helps learners prepare more intentionally for bedside activities and operative experiences.

**Fig. 1:**
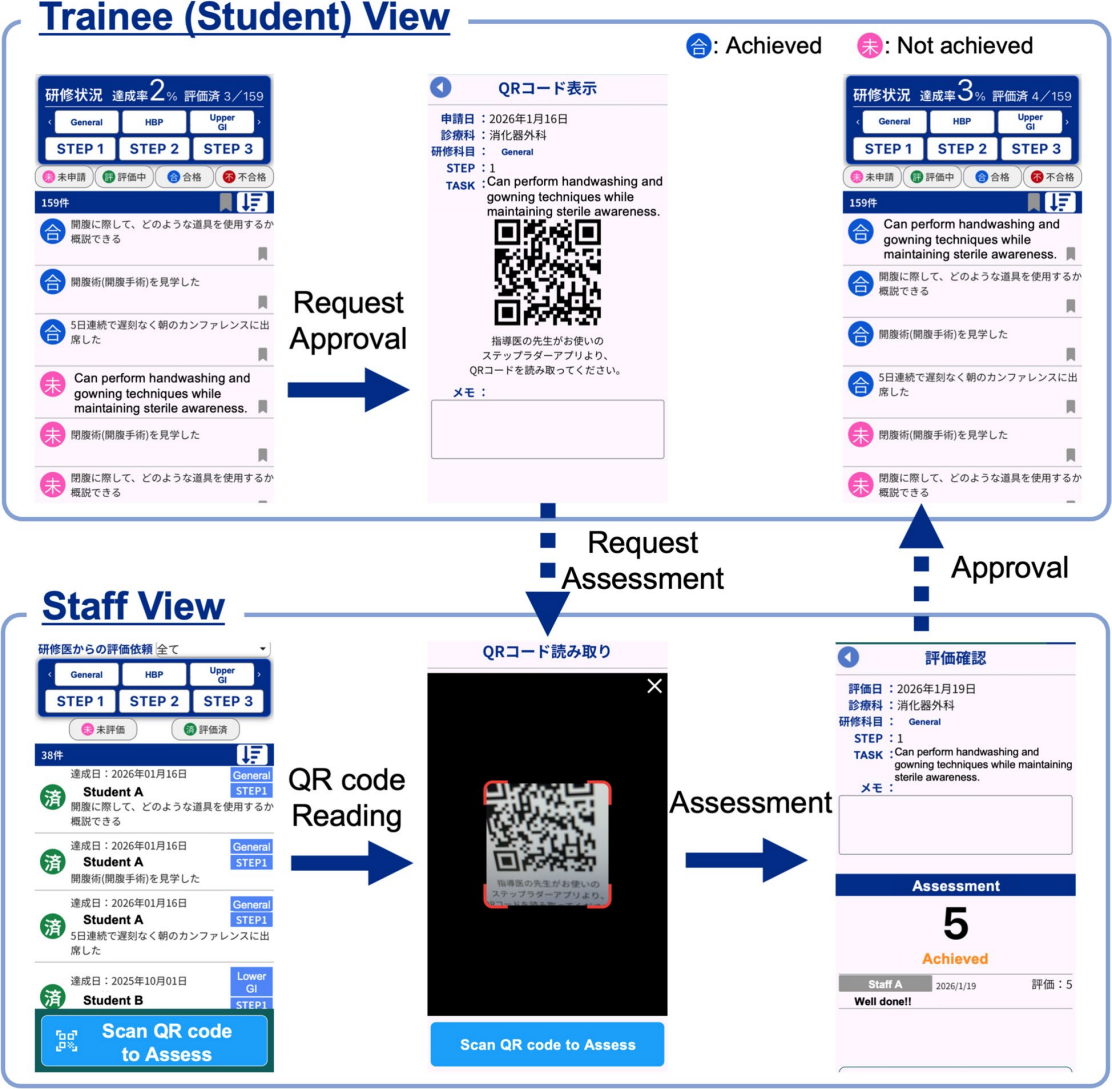
Step Ladder System application interfaces and assessment workflow. The upper panel shows the trainee (student) view, where tasks are organized by Step (Step 1–3) and clinical domain. After completing a task, the student submits a task assessment request by generating a QR code. The lower panel shows the staff view, where instructors scan the QR code, review the submitted task, and perform a task-level assessment and approval using a 5-point assessment scale. At our institution, an assessment score of 3 or higher is defined as task achievement, and tasks meeting this criterion are recorded as completed and reflected in the trainee’s progress dashboard

The SLS also included a progress dashboard that displayed each student’s current task status in a stepwise format, allowing any supervising faculty member to understand the learner’s level and verify task submissions. The SLS was originally implemented as a paper-based checklist in the Department of Obstetrics and Gynecology at Tottori University Hospital in June 2019 and was subsequently converted into a mobile application compatible with both iOS and Android devices, with app development commencing in April 2021 and routine educational use beginning in April 2022. Since its implementation, the mobile application has been adopted across multiple clinical departments, including surgical clerkships, well before the 2025 academic year. Students logged completed tasks through the application, and faculty members provided verification and feedback using the same platform (Fig. 1). Separate task modules are available for general surgery and for four subspecialty areas: upper gastrointestinal, lower gastrointestinal, hepatobiliary-pancreatic, and pediatric surgery.

For clarity to an international audience, selected interface elements directly related to the assessment process are presented in English, while non-essential user interface components remain in the original language.

All tasks included in the SLS were reviewed and finalized by faculty consensus in April 2025 (Version 1.7), and this version was used throughout the study period. The detailed SLS task lists (Japanese and English versions) have been deposited in figshare (10.6084/m9.figshare.30758705) [18].

### General Self-Efficacy Scale (GSES)

Students’ self-efficacy was measured using the GSES, a validated 16-item yes/no instrument frequently used in Japanese educational settings [16, 17]. Raw scores range from 0 to 16, with higher scores reflecting stronger self-efficacy. Responses were scored with the standardized 0/1 system, and total scores were converted into norm-referenced standardized scores based on the official table for Japanese student populations. The GSES has shown good internal consistency in previous studies (Cronbach’s α ≈ 0.80 in Japanese cohorts).

GSES data were collected at two time points—the first day (pre-test) and the last day (post-test) of the four-week clerkship—across both the retrospective and prospective phases of the study.

For students who rotated between April and May 2025, these GSES assessments had already been conducted as part of routine educational evaluation, and the resulting pre–post data were retrospectively included under institutional review board approval.

Starting from June 2025, the same assessment procedure was carried out prospectively after obtaining written informed consent. For all participants, pre- and post-responses were linked using anonymized alphanumeric identification codes. The linkage table was stored separately from the questionnaires, and all analytical datasets were fully anonymized prior to analysis. No missing data were observed.

### Step Ladder System (SLS) engagement

Engagement with the SLS was measured by the total number of task entries recorded in the mobile app during the clerkship. The same version of the SLS (Version 1.7), with identical task modules and user interface design, was used throughout the study. As a result, the total SLS input count provided a consistent and comparable measure of engagement across all participants, including both the retrospective and prospective cohorts.

Each entry reflected a two-step process:

1. Students submitted an entry and initiated a formal request for faculty evaluation through the mobile application after either acquiring prerequisite knowledge through preparatory learning or after observing and/or performing a clinical task, and determining that they were ready for assessment.
2. Faculty staff reviewed each submission and provided approval only when students demonstrated accurate knowledge acquisition, appropriate clinical reasoning, or the ability to perform the task safely under supervision. A total of 13 faculty members in the division of Gastrointestinal and Pediatric Surgery were involved as evaluators during the study period. Feedback was routinely provided to students through the mobile application and, when appropriate, verbally during clinical activities, in accordance with standard educational practice.

To enhance consistency across evaluators, SLS tasks were designed during the system development phase to allow binary judgment of task achievement (achieved/not achieved) based on clearly defined task descriptions, enabling comparable evaluations regardless of which faculty member performed the assessment.

Because both elements were automatically recorded in the app’s log system, the SLS input count provided an objective, uniform measure of engagement across students and clerkship cycles.

### Statistical analysis

Statistical analyses were performed using IBM SPSS Statistics for Mac, Version 27.0 (IBM Corp., Armonk, NY, USA). All graphical visualizations were generated using R (Version 4.5.2; R Foundation for Statistical Computing, Vienna, Austria). Continuous variables are presented as mean ± standard deviation (SD). Visual inspection of distributions for standardized GSES scores and ΔGSES values revealed no major deviations from normality, supporting the use of parametric methods. ΔGSES was defined as the difference between post- and pre-clerkship standardized GSES scores.

Changes in standardized GSES scores from pre- to post-clerkship were analyzed using a paired t-test, and the paired-samples effect size (Cohen’s d) was calculated. In addition, paired t-tests were conducted separately within the low and high baseline GSES subgroups, defined by dichotomizing baseline GSES scores at the median, to evaluate pre–post changes within each subgroup.

Directional tendencies in ΔGSES values (positive vs. negative change), excluding zero-change cases, were assessed using two-tailed binomial tests. This nonparametric method was selected because subgroup sample sizes were small, and the main goal was to see if positive self-efficacy changes happened more often than by chance, regardless of the change size. Binomial tests were performed separately for high and low SLS input groups and for high and low baseline GSES groups. The high/low SLS classifications used the median value as the cutoff for each variable.

Pearson’s correlation coefficient was used to assess linear relationships between continuous variables, in line with standard practice when variables are approximately normally distributed. A *p*-value < 0.05 was considered statistically significant for all analyses.

## Results

### Participant characteristics

During the study period, 24 sixth-year medical students completed the surgical clerkship and all required assessments (Table 1).

**Table 1:**
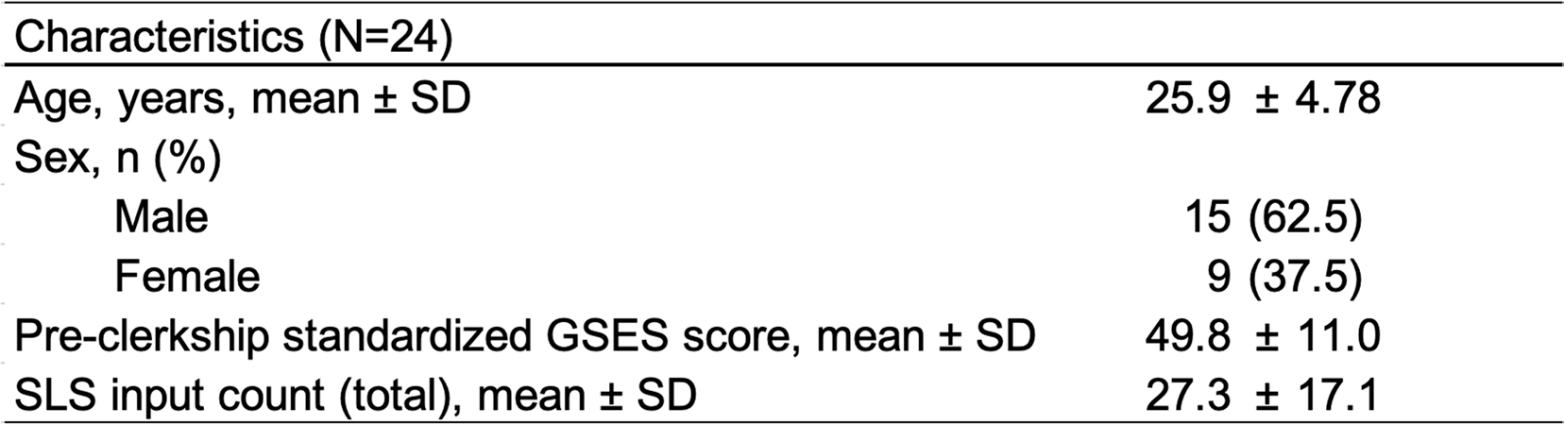
Baseline characteristics of study participants

*SD* Standard deviation, *GSES *General Self-Efficacy Scale, *SLS* Step Ladder System

The average age of participants was 25.9 ± 4.78 years, and 62.5% were male. Pre-clerkship standardized GSES scores showed significant variability among individuals (average 49.8 ± 11.0), indicating a diverse baseline level of general self-efficacy within the group. Engagement with the SLS also varied greatly among students, with a mean total SLS input of 27.3 ± 17.1 tasks. This wide range reflects considerable individual differences in SLS engagement.

### Changes in self-efficacy during the clerkship

Overall, standardized GSES scores increased significantly from pre- to post-clerkship (pre: 49.8 ± 11.0 vs. post: 53.8 ± 12.2; paired t-test, *p =* 0.009, Cohen’s d = 0.582, Table 2).

**Table 2:**
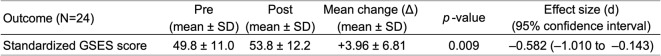
Changes in standardized GSES scores before and after surgical clerkship

Paired t-test was used for the pre-post comparison; d = Cohen's d (paired-samples effect size);* GSES* General Self-Efficacy Scale

**Fig. 2:**
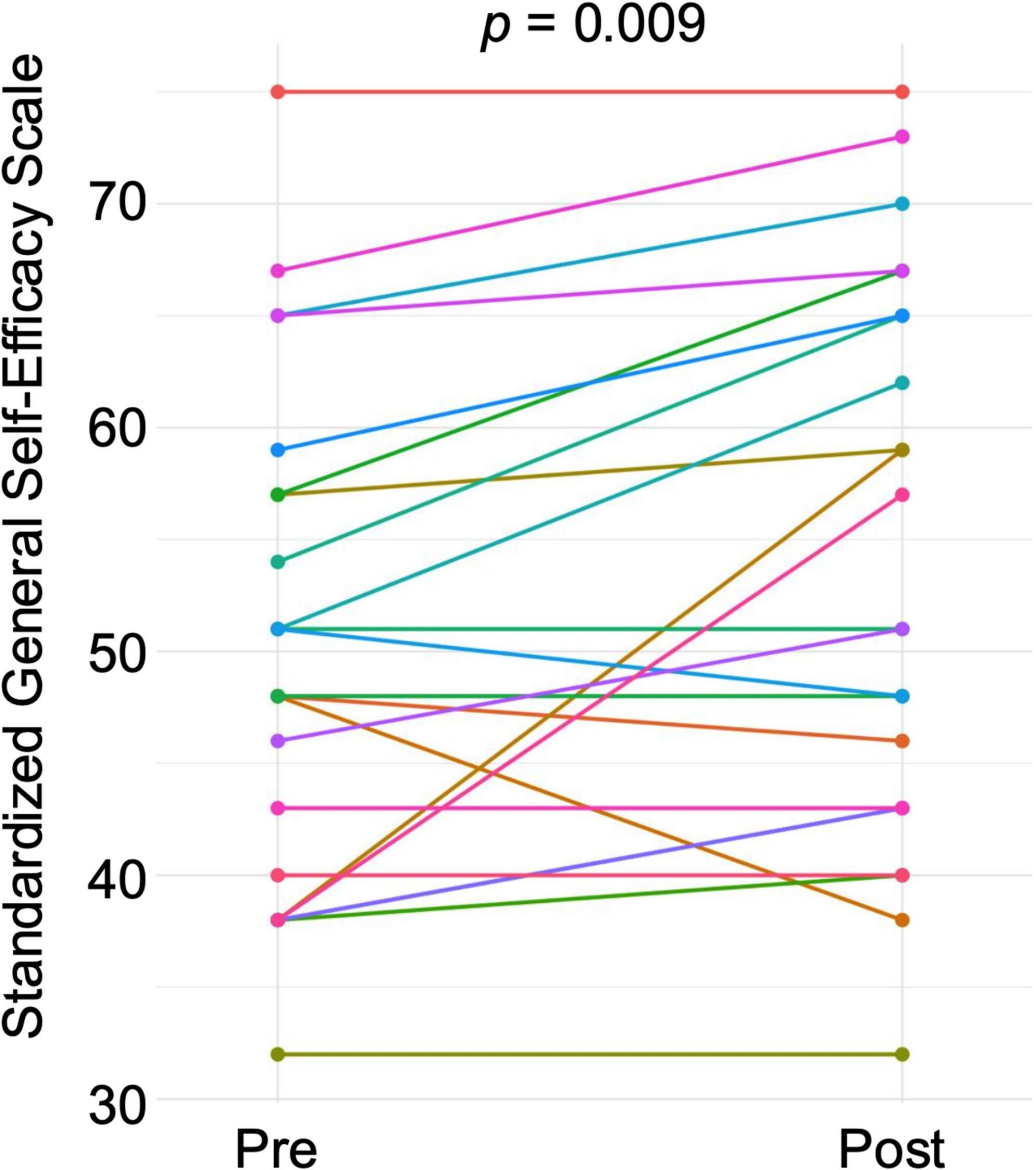
Individual trajectories of standardized GSES scores from pre- to post-clerkship. Each line represents the change in the standardized General Self-Efficacy Scale (GSES) score for an individual student (*N =* 24). Scores significantly increased from pre- to post-clerkship (paired t-test; Cohen’s d = 0.582)

Figure 2 shows the individual trajectories, indicating that although the amount of improvement differed, most students experienced a positive change. Only a few showed no change or slight declines.

### Directional analysis of changes by SLS engagement level

**Fig. 3:**
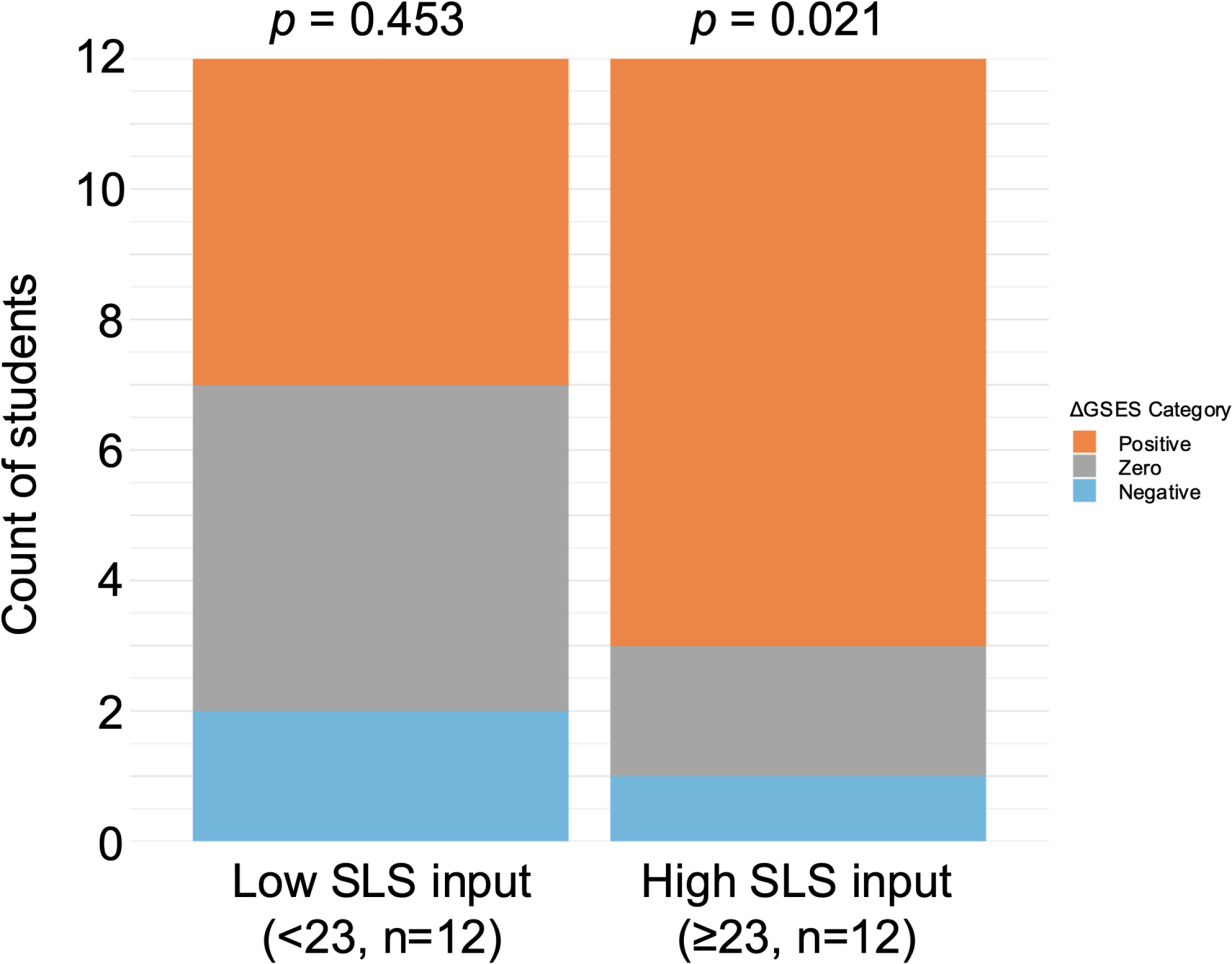
Distribution of positive, zero, and negative changes in standardized GSES scores (ΔGSES) according to SLS input level. Stacked bars show the number of students with positive, zero, and negative changes in standardized GSES scores. A binomial test excluding zero-change cases indicated a significant predominance of positive ΔGSES in the High-input group (*P =* 0.021), whereas no significant directional tendency was observed in the Low-input group (*p =* 0.453)

To further analyze the self-efficacy changes, ΔGSES values were grouped into positive, zero, or negative categories. When students were divided by SLS input level (median split), a clear pattern appeared (Fig. 3).

In the High SLS-input group, positive ΔGSES occurred significantly more often than negative ΔGSES (binomial test, *p =* 0.021). In contrast, the Low SLS-input group showed no significant trend (*p =* 0.453), with positive and negative changes happening roughly at chance levels.

### Directional analysis by baseline self-efficacy

**Fig. 4:**
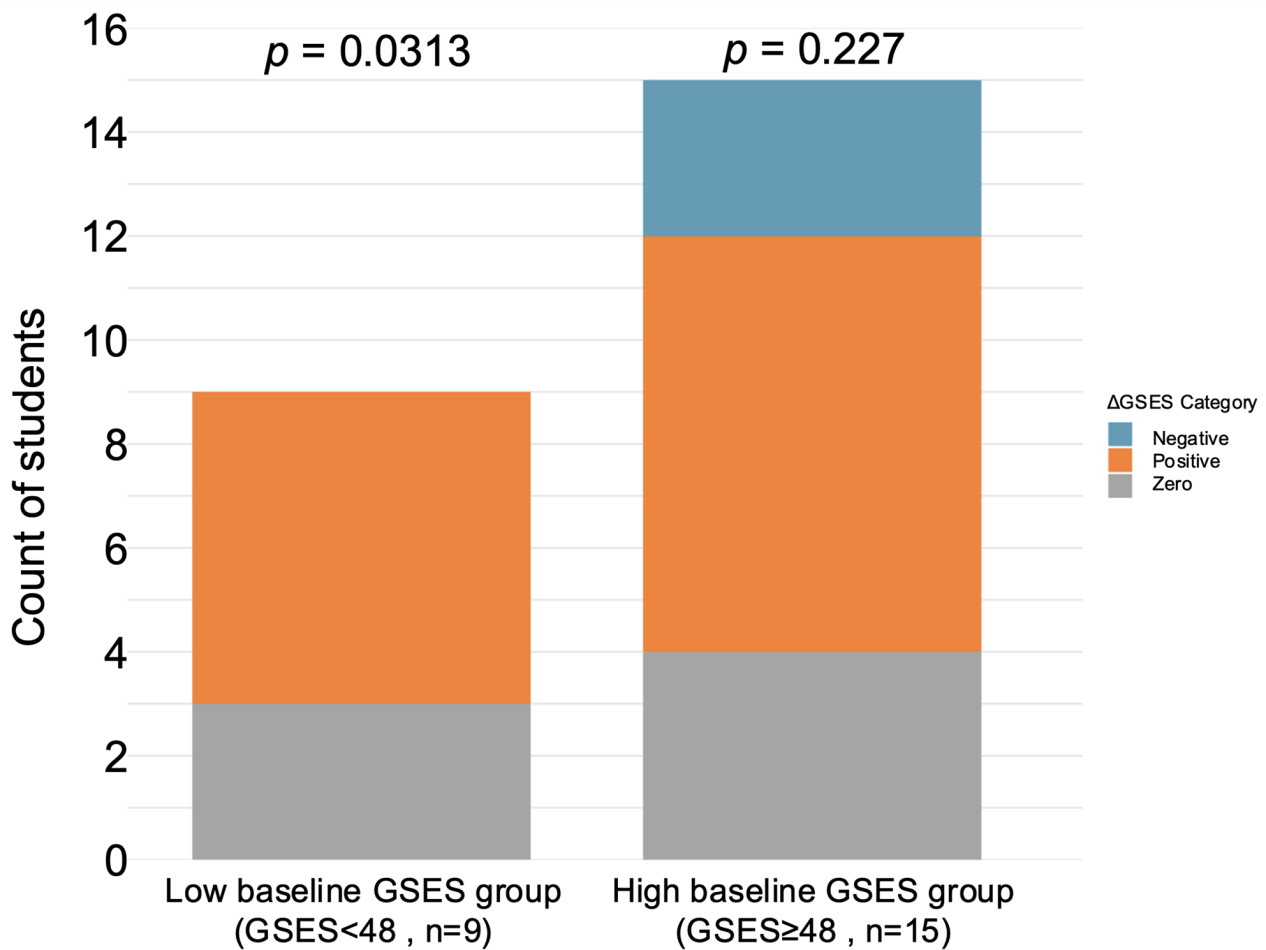
Distribution of ΔGSES directions by baseline GSES group

A similar analysis was performed after dividing students into low and high baseline GSES groups (Fig. 4).

Positive, zero, and negative changes in standardized GSES scores are shown for Low and High baseline groups. Binomial tests (zero excluded) indicated a significant predominance of positive ΔGSES in the Low baseline group (*p =* 0.031), whereas no significant directional bias was observed in the High baseline group (*p =* 0.227). Statistical test: Binomial test (excluding zero-change cases).

In the Low baseline GSES group, positive changes in ΔGSES occurred significantly more often than negative changes (binomial test, *p =* 0.031). In the High baseline GSES group, the distribution of positive and negative ΔGSES did not differ significantly from chance (*p =* 0.227).

### Association between SLS engagement and self-efficacy development

**Fig. 5:**
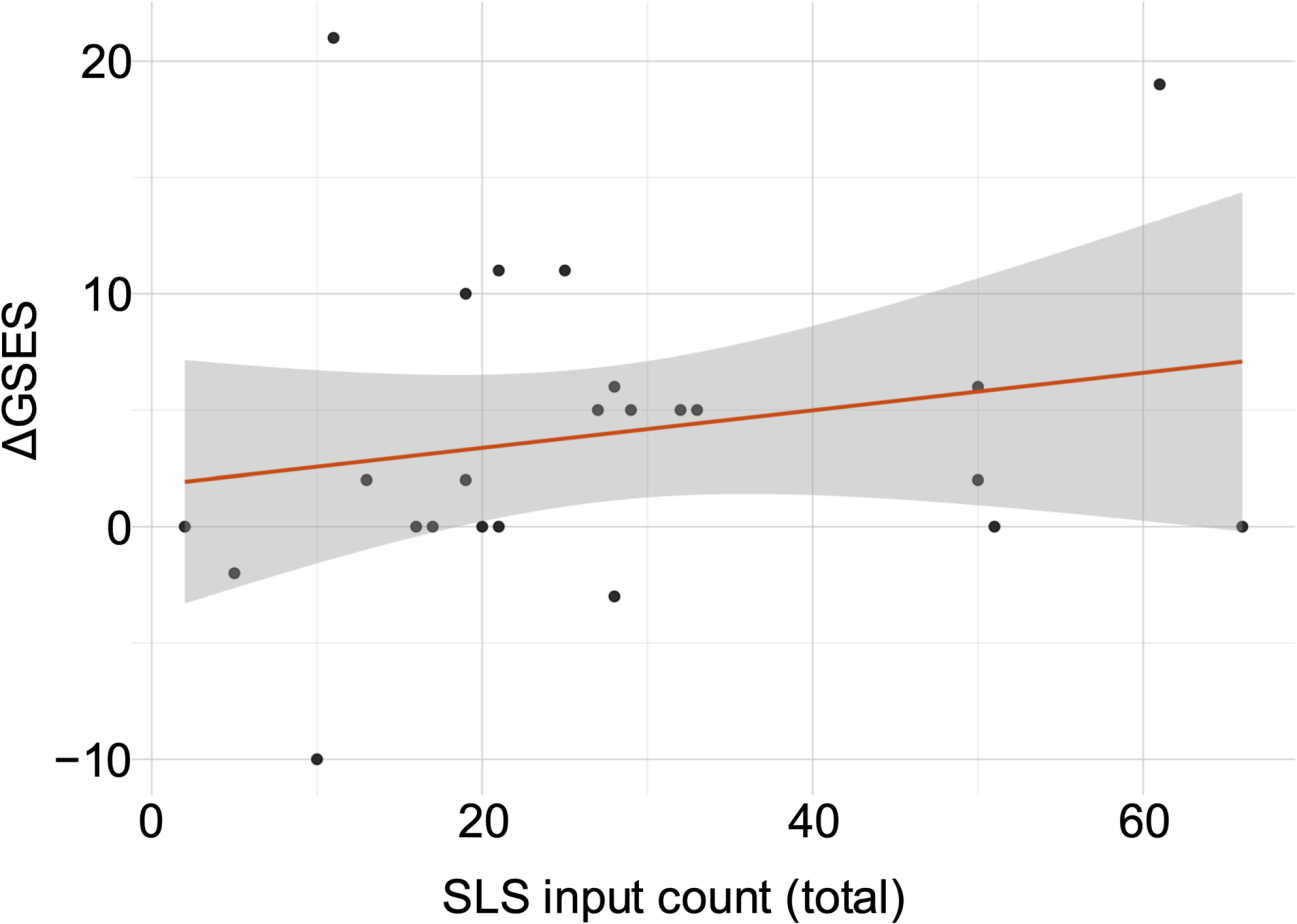
Scatterplot of SLS input count and change in standardized GSES score (ΔGSES)

To assess whether the amount of learning activity was linked to the level of self-efficacy improvement, Pearson’s correlation was calculated between total SLS input count and ΔGSES. A weak, non-significant positive correlation was found (*r =* 0.20, *p =* 0.34), as shown in Fig. 5. Although the scatterplot shows a slight upward trend, the wide spread around the regression line indicates considerable individual differences.

Although ΔGSES tended to increase with higher SLS input counts, the correlation was weak and did not reach statistical significance (Pearson's correlation, two-tailed, *r =* 0.20, *p =* 0.34). The gray band represents the 95% confidence interval around the fitted regression line.

## Discussion

In this study, we examined how implementation of the SLS, a structured learning support framework designed to make clinical expectations visible, was associated with medical students’ self-efficacy trajectories during a four-week surgical clerkship. Three principal findings emerged, indicating that lower baseline self-efficacy and greater engagement with the SLS were associated with more frequent positive directional shifts in self-efficacy.

First, students showed a statistically significant increase in standardized GSES scores from pre- to post-clerkship (Fig. 2). Importantly, the GSES is a domain-general psychological measure and does not directly assess task-specific or clinical competence. Accordingly, changes in GSES scores should be interpreted as reflecting learners’ perceived agency, confidence, or readiness for learning, rather than demonstrable acquisition of surgical competence. The moderate effect size suggests that the improvement in GSES is likely due to a meaningful educational impact rather than a statistical artifact of paired testing. This demonstrates that the clerkship experience, provided in the SLS-supported learning environment, was associated with measurable gains in self-efficacy. Second, students who engaged more actively with the SLS, measured by higher total input counts, exhibited a clearer trend toward positive changes in ΔGSES (Fig. 3). Although Pearson’s correlation analysis did not demonstrate a statistically significant linear association between SLS input volume and ΔGSES, directional analysis indicated that students with higher SLS engagement were significantly more likely to show positive changes in self-efficacy. These findings suggest that the relationship between SLS engagement and self-efficacy change is not simply dose-dependent. This does not dismiss the potential benefits of SLS participation but suggests that measurable changes in self-efficacy are affected by multiple factors beyond just task count, including the nature of clinical experiences, the quality of supervision, and students’ initial expectations. Third, the clerkship students with lower initial self-efficacy were especially likely to benefit from the experience, showing greater improvements than their higher-confidence peers (Fig. 4). This pattern suggests that the SLS may be particularly beneficial for learners who begin with less confidence. By promoting intentional preparatory learning, clarifying expected learning targets and expectations, and enabling structured documentation of progress, the SLS appears to support self-efficacy development in surgical training. Importantly, the SLS was not designed as a summative competency assessment or entrustment framework. Rather, it functions as a learner-facing scaffold to make expectations explicit, structure preparatory learning, and support reflection on clinical exposure. Progression through SLS steps therefore represents increasing engagement with learning tasks, not formal certification of workplace competence. Students with lower initial confidence, and those who engaged more actively with the SLS, seemed to benefit most from the system.

Taken together, these findings suggest that engagement with the SLS is associated with enhanced self-efficacy development during surgical training by encouraging intentional preparation, clarifying expectations, and allowing structured documentation of progress. The system seems particularly helpful for learners who start the clerkship with less confidence or who participate more actively in its tasks. Self-efficacy has long been recognized as a key determinant of motivation, persistence, and performance in medical education [1, 19, 20]. Learners with higher self-efficacy demonstrate greater willingness to participate actively in clinical tasks, to seek feedback, and to regulate their learning strategies [21]. Educational interventions that provide clear goals, promote mastery experiences, or enhance feedback have consistently been shown to strengthen self-efficacy in clinical environments [22, 23]. However, traditional surgical clerkships often place students in observer-dominant roles within hierarchical teams, limiting opportunities to internalize mastery experiences [24, 25]. In this setting, the mechanisms of the SLS, clarification of expected competencies, visualization of progress, and structured documentation of achievements, may help mitigate well-documented barriers to self-efficacy development. A notable implication of this study is the potential role of the SLS in promoting proactive learner preparation. Prior research indicates that medical students frequently struggle to determine what knowledge or skills they should acquire before participating in clinical rotations, particularly in surgery [26–29]. By presenting tiered, achievable tasks aligned with authentic clinical learning activities, the SLS made expectations explicit and allowed students to engage in intentional, goal-oriented preparation rather than passive exposure.

Such advance structuring likely enhanced students’ perceived control and goal-directed action; two core components of self-efficacy within social cognitive theory [30]. Furthermore, because SLS progress is visible to all supervising faculty, substitute instructors could immediately understand each student’s current level and provide consistent, level-appropriate guidance. This enhanced continuity of instruction is likely to support students’ sense of progression and foster their self-efficacy.

Despite these encouraging findings, several limitations should be acknowledged. First, the primary outcome (GSES) is a self-reported psychological measure. Increases in ΔGSES may not necessarily reflect parallel improvements in actual clinical competence. Incorporating objective performance assessments, such as OSCEs (Objective Structured Clinical Examinations), DOPS (Direct Observation of Procedural Skills), and workplace-based evaluations, would provide a more comprehensive assessment of the educational impact of the SLS. Self-efficacy was assessed only at the beginning and end of the four-week clerkship; therefore, changes in self-efficacy during intermediate phases of the rotation could not be examined. Second, a Hawthorne effect cannot be excluded. Because students were aware that their SLS activity and GSES responses were monitored, study participation itself may have enhanced attentiveness or motivation, thereby influencing the observed self-efficacy changes. Third, clinical exposure was not standardized across students. Although all participants completed the same departmental rotation, there was substantial variability in operative case assignments, procedural opportunities, and teaching styles among supervising faculty. These differences may have influenced both SLS engagement and GSES outcomes, making it difficult to isolate the effect of the SLS from other educational factors. Fourth, SLS input count functioned only as a quantitative proxy for engagement. This measure does not capture the difficulty, authenticity, or instructional quality of completed tasks, which may explain the modest correlation observed between total input count and ΔGSES. Fifth, baseline self-efficacy differences likely introduced ceiling effects. Students with high initial GSES scores had limited room for improvement, whereas those with lower initial self-efficacy demonstrated proportionally greater gains, influencing subgroup results. Finally, the study was conducted in a single department at a single institution with a limited sample size (*N =* 24). These factors restrict generalizability and reduce statistical power.

Nevertheless, the findings of this exploratory study indicate that the SLS is a promising educational tool for enhancing medical students’ self-efficacy during surgical clerkships. By making expectations explicit, enabling progress visualization, and facilitating consistent supervisory feedback, the SLS may support the shift toward more active, learner-centered clinical training environments. Future research should investigate the SLS across multiple departments and institutions, explore qualitative aspects of learner engagement, and examine long-term outcomes such as preparedness for residency and sustained self-regulated learning. In addition, future work will include psychometric evaluation of the SLS task hierarchy using Rasch analysis, as well as integration with objective performance assessments, such as OSCEs, to examine the relationship between task engagement, self-efficacy, and observable clinical performance. Given its feasibility and adaptability, the SLS has considerable potential as a scalable framework within competency-based medical education.

## Conclusion

This study demonstrated that medical students’ self-efficacy increased significantly during a four-week surgical clerkship. The SLS may contribute to this improvement by clarifying expected learning targets and expectations, supporting intentional preparation, and providing a structured mechanism for documenting progress. Although the quantitative association between total SLS input count and the magnitude of self-efficacy change was modest, students with lower baseline self-efficacy showed a clear tendency toward positive improvement. These findings suggest that the SLS may be particularly beneficial for learners who begin clerkships with limited confidence.

This exploratory single-institution study is constrained by its small sample size and reliance on self-reported outcomes. Nonetheless, the results indicate that the SLS is a feasible and promising framework for enhancing learner-centered surgical education. Future research should assess its generalizability in other clinical settings, incorporate objective performance measures, and examine its long-term influence on perceived preparedness for residency.

## Supplementary Information

Supplementary Material 1.

## Data Availability

The datasets generated and analyzed during the current study contain identifiable educational records and cannot be publicly shared due to institutional and ethical restrictions. De-identified data may be made available from the corresponding author on reasonable request and with approval from the Ethics Committee of Tottori University Faculty of Medicine.

## Abbreviations

SLS: Step Ladder System
GSES: General Self-Efficacy Scale
