# Supplementary material for: Climbing the ladder of confidence: effects of the step ladder system on medical students’ self-efficacy during a surgical clerkship
Source: BMC Med Educ. 2026 Apr 6;26:805. doi: 10.1186/s12909-026-09110-0 (PMC13217677; doi:10.1186/s12909-026-09110-0)
Supplement: Supplementary file 1 — Supplementary Material 1. [file 12909_2026_9110_MOESM1_ESM.pdf]

Supplementary material 3

Table 1

Standards for QUality Improvement Reporting Excellence in Education: SQUIRE-EDU

Text section and item name	SQUIRE item description	SQUIRE-EDU extension description
**Notes to authors**	The SQUIRE guidelines provide a framework for reporting new knowledge about how to improve healthcare.<br>The SQUIRE guidelines are intended for reports that describe system level work to improve the quality, safety, and value of healthcare, and used methods to establish that observed outcomes were due to the intervention(s).<br>A range of approaches exists for improving healthcare. SQUIRE may be adapted for reporting any of these.<br>Authors should consider every SQUIRE item, but it may be inappropriate or unnecessary to include every SQUIRE element in a particular manuscript.<br>The SQUIRE glossary contains definitions of many of the key words in SQUIRE.<br>The Explanation and Elaboration document provides specific examples of well-written SQUIRE items, and an in-depth explanation of each item.<br>Please cite SQUIRE when it is used to write a manuscript.	The SQUIRE-EDU extension of the SQUIRE guidelines provides a framework intended to increase the completeness, transparency, and replicability of published reports that describe systematic efforts to improve health professions education.<br>They apply to all learning settings (e.g., classroom, simulation, clinical, etc.).<br>The guidelines encourage the description of the process and context of educational change, use of iterative cycles, and use of data over time.<br>Authors should consider every SQUIRE and SQUIRE-EDU item, but it may be inappropriate or unnecessary to include every SQUIRE and SQUIRE-EDU element in a particular manuscript.<br>Not all items have an EDU extension. If there is no EDU extension, use the SQUIRE item. If there is an EDU extension, it may be used on its own or in conjunction with the SQUIRE item.<br>Educators use a range of systematic methods to make education and healthcare demonstrably better. SQUIRE-EDU may be adapted for reporting any of these methods.<br>Please cite SQUIRE-EDU when it is used to write a manuscript.
**Title and abstract**		
1. Title<br>See P1L1-2.	Indicate that the manuscript concerns an initiative to improve healthcare (broadly defined to include the quality, safety, effectiveness, patient-centeredness, timeliness, cost, efficiency, and equity of healthcare)	**EDU 1:** Indicate that the manuscript concerns efforts to improve health professions education systems and learning
2. Abstract<br>See P2-3L24-53.	a. Provide adequate information to aid in searching and indexing<br>b. Summarize all key information from various sections of the text using the abstract format of the intended publication or a structured summary such as: background, local problem, methods, interventions, results, conclusions	**EDU 2:** Keywords include a focus on education and learning
**Introduction: Why did you start?**		
3. Problem description<br>See P5L94-98.	Nature and significance of the local problem	**EDU 3:** Description of the nature and significance of the need for change in the local educational system
4. Available knowledge<br>See P4-5L70-98.	Summary of what is currently known about the problem, including relevant previous studies	—
5. Rationale<br>See P4L83-85,P5L87-89, P18L373-378.	Informal or formal frameworks, models, concepts, and/or theories used to explain the problem, any reasons or assumptions that were used to develop the intervention(s), and reasons why the intervention(s) was expected to work	**EDU 5:** Identify the guiding theory (learning, change, implementation, or other) and how it aligns with the need for change in the local educational system
6. Specific aims<br>See P5L10 1-10 5.	Purpose of the project and of this report	—
**Methods: What did you do?**		
7. Context<br>See P6L110-122.	Contextual elements considered important at the outset of introducing the intervention(s)	**EDU 7a:** Contextual elements for learning (e.g., setting, program, people, resources, social, geopolitical influences) before the intervention(s)<br>**EDU 7b:** The interrelationships between the contextual elements and the local educational and healthcare systems before the intervention(s)
8. Intervention(s)<br>See P8L159-161, P8-9L169-180.	a. Description of the intervention(s) in sufficient detail that others could reproduce it<br>b. Specifics of the team involved in the work	**EDU 8a:** Description of the primary interventions and co-interventions (e.g., faculty or tool development)<br>**EDU 8b:** Specify how the interprofessional education team (e.g., faculty, staff, patients, and learners) was part of the design of the intervention
9. Study of the intervention(s)<br>See P7L131-133, P10L201-206, P11L225-239.	a. Approach chosen for assessing the impact of the intervention(s)<br>b. Approach used to establish whether the observed outcomes were due to the intervention(s)	**EDU 9a:** Approach used to understand the impact of the educational intervention(s) on the learner and beyond, such as impact on patients, families, the community, faculty, educational program, or the healthcare system<br>**EDU 9b:** Approach to assess the fidelity of and the iterative changes to the planned intervention(s) over time

(*Table continues*)

Table 1

(Continued)

Text section and item name	SQUIRE item description	SQUIRE-EDU extension description
10. Measures<br>See P9L182-188, P10L201-206.	a. Measures chosen for studying processes and outcomes of the intervention(s), including rationale for choosing them, their operational definitions, and their validity and reliability<br>b. Description of the approach to the ongoing assessment of contextual elements that contributed to the success, failure, efficiency, and cost<br>c. Methods employed for assessing completeness and accuracy of data	**EDU 10:** Quantitative and/or qualitative measures chosen to assess the educational processes and outcomes on learners, faculty, educational programs, patients, families, healthcare systems, or communities
11. Analysis<br>See P11L218-239.	a. Qualitative and quantitative methods used to draw inferences from the data<br>b. Methods for understanding variation within the data, including the effects of time as a variable	—
12. Ethical considerations<br>See P12-13L242-265.	Ethical aspects of implementing and studying the intervention(s) and how they were addressed, including, but not limited to, formal ethics review and potential conflict(s) of interest	**EDU 12:** Approaches to address vulnerability of learner participants
**Results: What did you find?**		
13. Results<br>See P14-16L284-337, P10L199.	a. Initial steps of the intervention(s) and their evolution over time (e.g., time-line diagram, flow chart, or table), including modifications made to the intervention during the project<br>b. Details of the process measures and outcome<br>c. Contextual elements that interacted with the intervention(s)<br>d. Observed associations between outcomes, interventions, and relevant contextual elements<br>e. Unintended consequences such as unexpected benefits, problems, failures, or costs associated with the intervention(s)<br>f. Details about missing data	**EDU 13a:** For each educational intervention and co-intervention, provide details about iterative modifications based on the assessment of the learning
**Discussion: What does it mean?**		
14. Summary<br>See P16-17L338-352, P17L353-357.	a. Key findings, including relevance to the rationale and specific aims<br>b. Particular strengths of the project	**EDU 14:** Connect the findings to the guiding theory (learning, change, implementation, other) used to direct the change in the local educational system
15. Interpretation<br>See P16-17L345-356.	a. Nature of the association between the intervention(s) and the outcomes<br>b. Comparison of results with findings from other publications<br>c. Impact of the project on people and systems<br>d. Reasons for any differences between observed and anticipated outcomes, including the influence of context<br>e. Costs and strategic trade-offs, including opportunity costs	**EDU 15c:** Include the impact of the intervention(s) on learners, faculty, educational program, patients, families, healthcare systems, or communities
16. Limitations<br>See P18-19L380-401.	a. Limits to the generalizability of the work<br>b. Factors that might have limited internal validity such as confounding, bias, or imprecision in the design, methods, measurement, or analysis<br>c. Efforts made to minimize and adjust for limitations	—
17. Conclusions<br>See P19-20L412-419.	a. Usefulness of the work<br>b. Sustainability<br>c. Potential for spread to other contexts<br>d. Implications for practice and for further study in the field<br>e. Suggested next steps	**EDU 17b:** Scalability of the work to other learners and contexts<br>**EDU 17d:** Lessons learned for clinical practice, education, and policy
**Other information**		
18. Funding<br>See P13L271-272.	Sources of funding that supported this work. Role, if any, of the funding organization in the design, implementation, interpretation, and reporting	—
